# Comparative Study of Square and Circular Loop Frequency Selective Surfaces for Millimeter-Wave Imaging Diagnostics Systems

**DOI:** 10.3390/s18093079

**Published:** 2018-09-13

**Authors:** Wahab Mohyuddin, Dong Hwi Kim, Hyun Chul Choi, Kang Wook Kim

**Affiliations:** School of Electronics Engineering, Kyungpook National University, 80 Daehak-ro, Buk-gu, Daegu 41566, Korea; chwahab@ee.knu.ac.kr (W.M.); eastsine766@gmail.com (D.H.K.); hcchoi@ee.knu.ac.kr (H.C.C.)

**Keywords:** frequency selective surface (FSS), millimeter-wave, imaging diagnostics systems, large-size FSS

## Abstract

A design method of large-sized square-loop and circular-loop frequency selective surface (FSS) filters for protection of mm-wave imagining receivers is presented. Due to fine cell structure requirements, the performance of the FSS structures at mm-wave frequencies can be significantly affected by fabrication tolerances, especially involved with large-size panel fabrication. Through a comprehensive parametric variation study on the performance of square-loop and circular-loop FSS structures, it is found that the circular-loop FSS structure performs much less sensitively to the fabrication tolerances, thereby producing better and consistent performances with given design values. As a design example, square-loop and circular-loop notch filters resonating at 105 GHz were designed and the performances were evaluated with multiple prototypes. The resonant frequencies of the implemented circular-loop FSS filters deviated by only about 0.5 GHz from the accurate designed value, which can be easily adjusted in the design process. The implemented square-loop and circular loop FSS filters provided low-loss in the pass-band and high rejection of 23 dB at the resonant frequency with good oblique angle performance.

## 1. Introduction

A frequency selective surface (FSS) consists of an array of identical metallic structures arranged as a single or multi-dimensional periodic surface. The periodic structures have extensively been used in series of microwave, millimeter-wave (mm-wave) and terahertz (THz) applications such as band-stop filters, dichroic reflectors and circuit absorbers [1]. When excited by an incident plane wave, the FSS resonates at a particular frequency or multiple frequencies depending on the properties of the periodic array structure, dimensions and dielectric substrate. The conventional FSSs are interpreted as capacitive or inductive structures or sometimes a combination of both [2,3,4,5,6].

A plethora of FSS configurations have been actively investigated in wide application areas such as reflectors for satellite communications [7,8], microwave radomes [9], waveguide filters [10], phase shifting surfaces [11], linear to circular polarization convertors [12,13] and various antennas [14]. In addition, the FSS has been used as metamaterials and metasurfaces in the mm-wave and THz technology for designing absorbers, sensors, spatial filters and imaging system components [15,16,17,18,19,20,21]. The FSS structures used in these studies include square structure, circular structure and the combination of square or circular with other structures.

Recently, the FSSs were implemented as notch, band-stop and high-pass filters for mm-wave imaging diagnostic systems [22,23,24,25,26]. In the studies, the resonant frequencies of the FSS-based filters were 105 GHz, 110 GHz, 140 GHz and 170 GHz for imaging diagnostic systems for tokamak plasmas [6,24]. In these systems, the FSS-based protection was essential to protect the receiver electronics from unwanted stray signals by providing high rejection of, often, powerful unwanted signals. For example, in the ECEI (electron cyclotron emission imaging) system, which extracts images of plasma temperature fluctuations in tokamak plasmas by detecting weak plasma radiated signals in mm-wave band, protecting Schottky diode detector arrays from high-power stray ECRH (electron cyclotron resonance heating) signals was required. A large-sized thin planar FSS filter was required to be placed in front of the detector array box to reject or weaken the powerful stray signal [26].

For mm-wave applications, the performance of the reported mm-wave FSS structures was highly dependent on the fabrication accuracy due to the fine cell structure requirements. Therefore, for the implementation, high-precision and expensive fabrication techniques such as Electro-Fine Forming (EF2) technology [23], thin-film technology [27] and microfabrication [28] were used to fabricate FSS filters. With small-sized FSS structures [27,28,29], high-precision fabrication technique can be applied without difficulty, but, as the size of the FSS panel becomes bigger, significant increase in fabrication cost can occur. In this regard, recently, our group has developed and reported a low-cost fabrication technique for large-size FSSs (~19.7 × 6.3 inches at 170 GHz) with high performance at mm-wave range to protect the mm-wave ECEI receiver [6]. In the study, an array of square loops on both sides of the substrate was used, but, with using the commercial PCB process, the resonant frequencies of the FSSs from multiple prototypes showed variations of around 3 GHz deviated from the designed values.

In this paper, a comprehensive comparative parametric analysis including the effect of fabrication tolerances on the performance for square- and circular-loop FSS structures at the millimeter-wave frequency range is presented. It is shown that an array of circular loops is found to produce more predictable and accurate results as compared with the array of square loops under the same fabrication tolerance conditions. To the best of authors’ knowledge, the configurations of circular-loop FSSs have not been yet employed previously in the mm-wave imaging systems.

## 2. Configuration of FSSs

**Figure 1:**
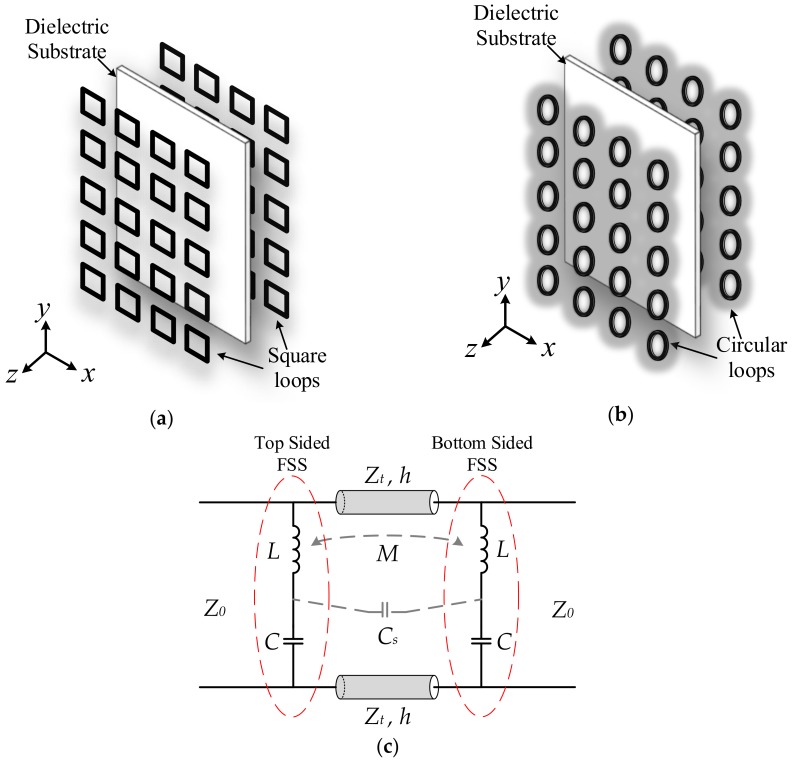
Single-layer double-sided FSSs: (**a**) Layout of a square-loop structure, (**b**) Layout of a circular-loop structure; (**c**) Equivalent circuit model.

With the proposed design of square-loop and circular-loop FSSs, periodic structures are comprised of an array of square loops and circular loops printed on both top and bottom sides of the supporting substrate. Figure 1a shows the layout of the square-loop FSS and the structure of the circular-loop FSS is shown in Figure 1b. The Roger 4003 substrate with loss tangent of 0.0027 and relative permittivity of 3.38 is used for the design of the proposed FSSs. The thickness of the dielectric substrate and metal thickness are chosen as 12 mil and 0.7 mil, respectively. Top and bottom sides of square-loop and circular-loop structures are symmetric as shown in Figure 1a,b.

The FSS is interpreted as capacitive or inductive structures or sometimes a combination of both. Therefore, the FSS can be modelled as an equivalent circuit of *L-C* networks. For both square-loop and circular-loop array structures, the equivalent circuit model (ECM) can be identical and is shown in Figure 1c. The ECM is a combination of two series *L-C* networks and mutual coupling components for the top and bottom sides of the substrate. The substrate is modelled as a short transmission line, which has a thickness *h*. When a perpendicularly polarized plane wave is impinged at the FSS, the capacitance is formed between gaps of the adjacent loops of the conducting material due to electric charge accumulation and the inductance occurs due to a flow of current around the adjacent loops. These electric charge densities are accumulated at the horizontal (*x*-direction) strips of the loops, creating the capacitive part “*C*.” Whereas, the magnetic field is induced around the vertical (*y*-direction) strips of the loops due to the flow of electric current that forms of an inductive part “*L*” in the ECM. Further, an electromagnetic interaction occurs between the top and bottom FSSs separated by the thin substrate, creating the mutual induction “*M*” and series capacitance “*C_s_*” in the ECM [6].

## 3. Parametric Simulations

The performances of the proposed FSS filters were simulated and optimized with the CST Microwave Studio^®^ (MWS) using the FSS unit cell (frequency domain) solver. By utilizing the CST’s Floquet-mode ports, the FSS structures were simulated as an infinite array of the unit cell of square and circular loops in CST MWS. The boundary conditions were set as unit cell boundary conditions and the meshing was set as tetrahedral spatial meshing.

**Figure 2:**
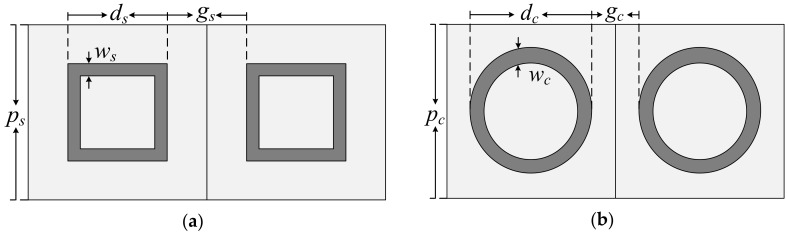
Dimension parameters: (**a**) Square-loop structure, (**b**) Circular-loop structure.

The frequency response of the FSS is determined by the structure shape and its dimension parameters. The key dimension parameters that largely affect the performance of the FSS filters are the loop length “*d*” (loop side length for the square-loop and outer diameter for the circular-loop), the loop width “*w*,” the unit cell period “*p*” and the gap between the loops “*g*” as shown in Figure 2a,b. For the parameters of square and circular loops, the subscripts “*s*” and “*c*” represent the square and circular structures, respectively, as shown in Figure 2.

**Figure 3:**
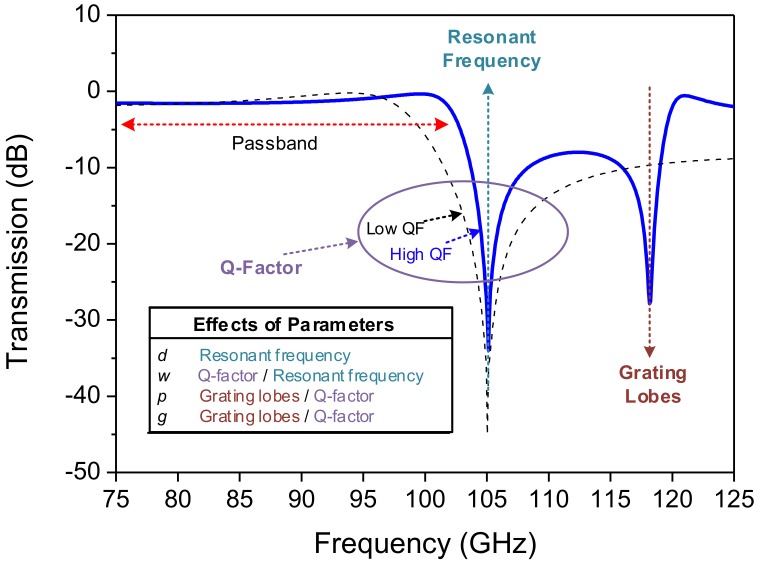
Typical characteristics of an FSS filter with effects of design parameters.

A typical frequency response of the loop-type FSS including the effects of the oblique angle incidence of a plane wave is shown in Figure 3. The main and accompanying effects of the aforementioned parameters (*d*, *w*, *p*, *g*) are also summarized. By adjusting these design parameters, the following FSS properties can be selected: resonant frequency, quality factor (Q-factor) and the frequency where grating lobes occur.

**Figure 4:**
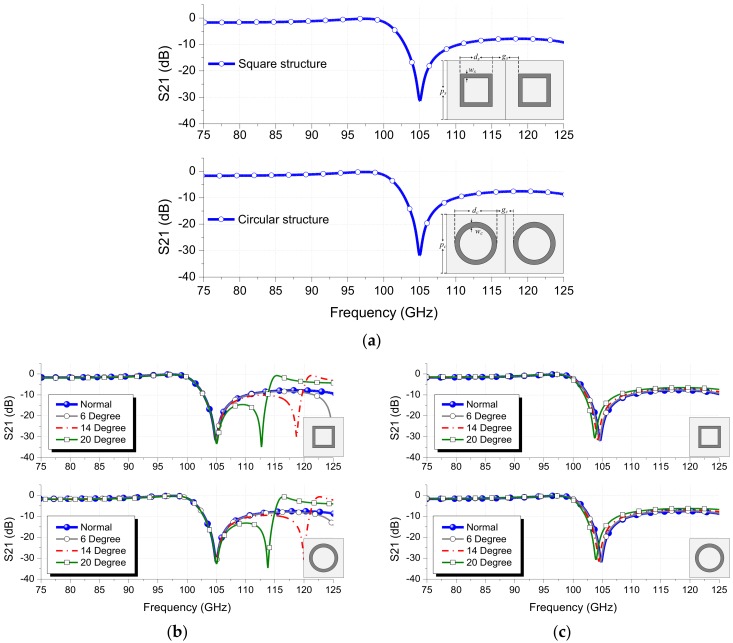
Frequency responses of square-loop and circular-loop FSSs: (**a**) normal incidence, (**b**) TE excitation, (**c**) TM excitation.

As for a design example, the FSS parameters are optimized for square-loop and circular-loop FSS structures to have the resonant frequency of 105 GHz as shown in Figure 4a. The design parameters selected for square-loop and circular-loop FSSs are *d_s_* = 23, *d_c_* = 26.9, *w_s_* = 4, *w_c_* = 4, *p_s_* = 60, *p_c_* = 60, *g_s_* = 37, *g_c_* = 33.1 (units: mil). It can be seen that both square- and circular-loop structures possess very similar frequency responses at the normal incidence of a plane wave for both TE and TM excitations. However, in the case of the oblique angular incidence, a slightly downward shift in the resonant frequency is observed for the TM excitation with no occurrence of grating lobes as compared with the TE excitation within the incidence angle of 20 degrees up to the frequency range of 125 GHz. Within 20 degrees of angular incidence, the shifts in the resonant frequency of the square-loop FSS were 0.1 GHz and 0.72 GHz for TE and TM polarizations, respectively. However, the shifts in the resonant frequency of the circular-loop FSS were 0.09 GHz and 0.88 GHz for TE and TM polarizations, respectively, as shown in Figure 4b,c. The effects of the design parameters on the frequency responses of the square- and circular-loop FSSs are described as the following.

**Figure 5:**
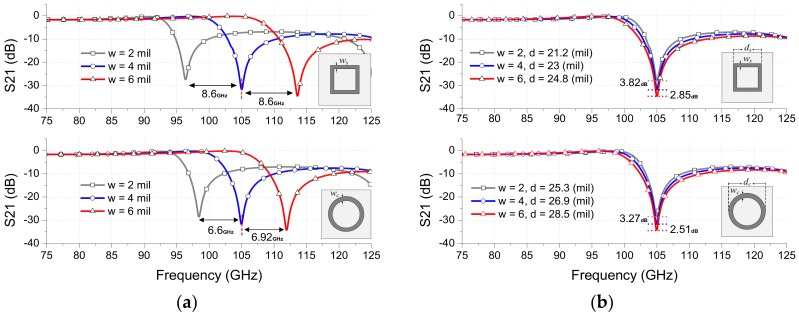
Effects of the loop width *w* on the loop-type FSSs: (**a**) Fixing *d*, *p* and *g* parameters, (**b**) Adjusting *d* with fixed *p* and *g*.

In the design, firstly, the loop width “*w*” should be chosen, since *w* strongly affects both the resonant frequency and the Q-factor of the FSS filter as shown in Figure 5. Figure 5a shows the variation of resonant frequencies as changing *w* with fixing *d*, *p* and *g* parameters. When *w* is increased, the inductance of the loop becomes smaller and thereby the resonant frequency is significantly increased with some improvement of rejection level. The Q-factor of the FSS filter, however, is decreased with the increase of *w* as shown in Figure 5b, which shows the FSS frequency responses by changing *w* and adjusting *d* with fixing *p* and *g* parameters to have the resonant frequency of 105 GHz. As can be seen, the higher Q-factor from the FSS filter can be obtained with a smaller value of *w*, which can be often limited by the fabrication tolerance. Therefore, *w* can be chosen to satisfy the low-loss passband requirement and the Q-factor. In this design, *w* is chosen as 4 mil by considering the used chemical etching fabrication condition.

**Figure 6:**
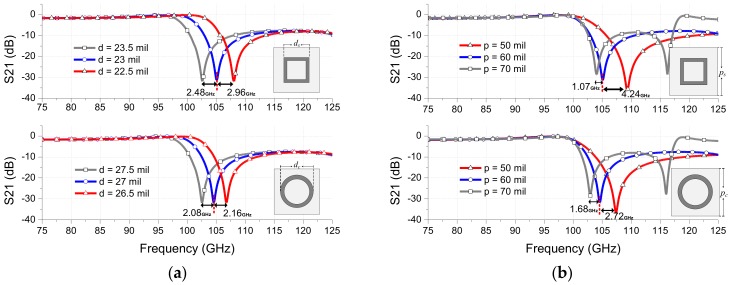

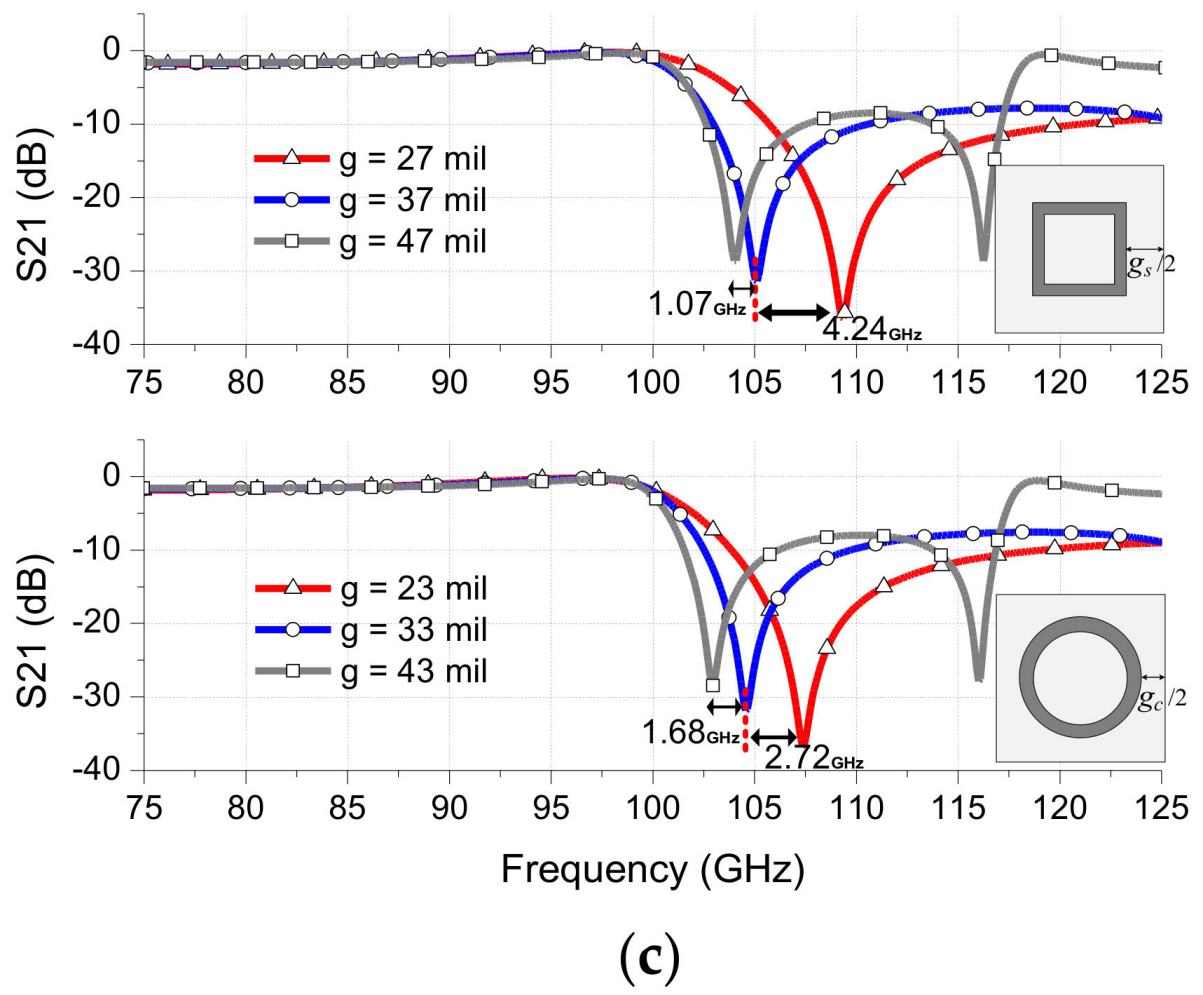
Influence of the design parameters on the FSS frequency response: (**a**) loop length “*d*,” (**b**) unit cell period “*p*,” (**c**) gap between the loops “*g*.”

Figure 6 shows the frequency response variations related to other parameters (*d*, *p* and *g*). With the selected *w*, the resonant frequency of the loop-type FSS filters is determined by the loop length “*d*.” As shown in Figure 6a, a change of the loop length *d* significantly shifts the resonance frequencies of both square- and circular-loop cases. A small decrease of 0.5 mil in *d* shifts the resonant frequencies upwards by 2.96 GHz and 2.16 GHz for square- and circular-loop FSSs, respectively. Further theoretical details regarding the effect of *d* on the resonant frequency were elaborated in Reference [30].

The main effect of choosing *p* (period of the unit cell) and *g* (gap between the loops) values is determination of the frequency where grating lobes occur as shown in Figure 6b,c. As values of *p* or *g* become smaller, the frequency of the grating lobes occurs farther from the resonant frequency. Also, similarly as *w*, the gap *g* between loops also affects the Q-factor. The higher Q-factor can be achieved by increasing the *g*, but, as a tradeoff, it results in producing closer grating lobes from resonant frequency, thereby lowering oblique incident angle performance. Increase of 10 mil in *p* or *g* results in the higher Q-factor but decreases the notch-rejection by 3.3 dB. It also causes the grating lobes to occur very closer to the resonant frequency. When the oblique incidence angle performance of the FSS is important, the gap between adjacent elements *g* should be less than half of the free space wavelength to avoid the scattering of the signal. However, for the normal incidence, the gap *g* can be equal to or less than one wavelength [30,31]. Though there exist the distinct main influences of the aforementioned parameters on the frequency response of the FSS, however, they all contribute more or less in the shift of the FSS resonant frequency. Furthermore, the variations in the dielectric properties (i.e., loss tangent and dielectric constant) of the substrate can slightly influence in the frequency shift. It is known that the frequency increase results in decreasing the dielectric constant of the substrate, which can shift the resonant frequency upwards. The change in the loss tangent also effects on the level of the notch-rejection.

## 4. Prototypes and Validation

The Rogers RO4003 substrate with thickness of 12 mil was chosen for design and fabrication of the proposed FSS filters. A commercial chemical etching (or PCB) process, which is a cost-effective process, was used for the fabrication. Required high-performance of the FSSs can be attained with careful optimization of dimension parameters even in the presence of fabrication tolerances involved with the low-cost commercial etching process. The commercial chemical etching process for printed circuits is typically performed as following. Firstly, the substrate is cleaned for lamination. Then, a photosensitive resist is laminated on the top and bottom sides of the substrate using hot-roller lamination process. A stencil film for square- and circular-loop structures is coated on the both sides of substrate and is exposed to the ultra-violet light, which hardens the structure patterns into acid resistance surfaces. Then, an etchant is sprayed on the substrate dissolving the exposed metal, which results in the etching the square- and circular-loop structures.

**Figure 7:**
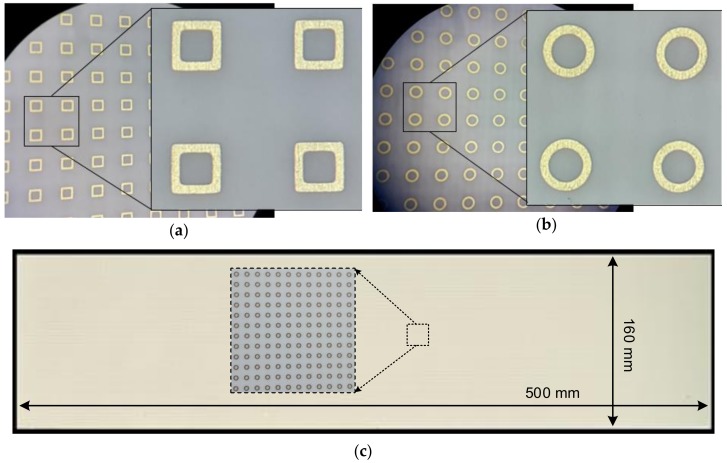
Fabricated FSS filters: (**a**) Square-loop structure, (**b**) Circular-loop structure, (**c**) Fabricated size of the FSS filters is 19.7 × 6.3 inches (500 × 160 mm).

Figure 7 shows the fabricated single-layer double-sided square and circular-loop FSS filters. The front and back configurations of the FSS filters are identical. The total size of the fabricated FSS panel is 19.7 × 6.3 inches (500 × 160 mm) for both cases, which is the required size to protect the detector array box in the ECE imaging system.

From Figure 7, it is observed that the fabricated square loops do not maintain their sharp corner edges and also the width of the loops is slightly irregular due to fabrication inaccuracy. It is also seen that the square-loops are not perfectly aligned: that is, some loops are tilted to left side and others are slanted to right side. Whereas, in the circular-loop array, there are no sharp corner edges and even if the loops are tilted, they remained aligned due to the circular property. The total number of elements in the fabricated square-loop or circular-loop FSSs are 128,905 (635 × 203). Therefore, for the measurements, arbitrary portions from the multiple fabricated FSS samples were selected and measured for the dimensions of the fabricated loops using an automatic CNC Vision Measurement machine. The comparison between the designed and fabricated values of the dimension parameters is shown in Table 1. It is obviously observed that the errors between the designed and fabricated parameter values in the case of the square-loop structure are much higher than those in the circular-loop structure: that is, the percentage errors of *d_s_* and *d_c_* are 1.46% and 0.07%, respectively. Under the same fabrication process with given fabrication tolerances, it can be seen that much smaller errors occurs in fabricated dimension parameters in the case of the circular-loop structures as compared with the square-loop structures, thereby expecting more predictable and accurate FSS performance with the circular-loop structures.

**Figure 8:**
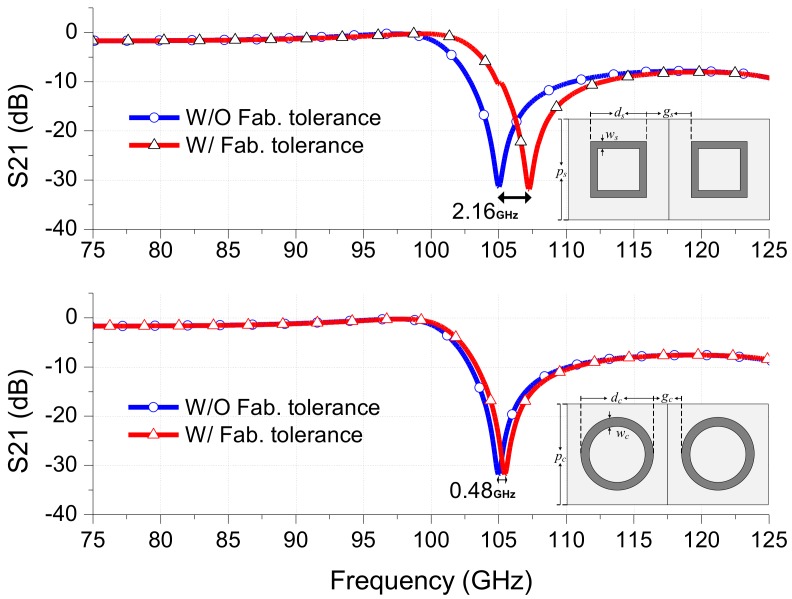
Influence of fabrication tolerances on the FSS frequency response.

Based on the measured parameter values, the influences of deviated parameter values due to the fabrication tolerances on the frequency responses of square and circular-loop FSSs are evaluated. Figure 8 shows the simulated results of the square-loop and circular-loop FSSs without and with consideration of fabrication tolerances. In Figure 8, in order to predict the possible shifts in the resonant frequencies of square- and circular-loop FSSs, the amount of averaged dimensional deviations due to fabrication tolerances for the square and circular loops shown in Table 1 are added to the optimized dimension parameters: that is, *d_sa_* = 23.65, *d_ca_* = 27.04, *w_sa_* = 3.75, *w_ca_* = 4.21, *p_sa_* = 60.06, *p_ca_* = 60.1, *g_sa_* = 36.41, *g_ca_* = 33.06 (units: mil). The subscripts “*sa*” and “*ca*” represent the square and circular structures parameters with addition of the averaged dimensional deviations. It is observed that the shift in resonant frequency of the square structure is around 2.16 GHz, whereas, for the circular structure it is just around 0.48 GHz. It is obvious that the shift in the resonant frequency is much smaller in the case of the circular-loops than the square-loops. Therefore, under the condition of non-negligible fabrication tolerances, the FSS structure with an array of circular loops produces less sensitive, more predictable and accurate results than the square-loop structures, especially when a large-size FSS structure to cover mm-wave frequency range is required.

## 5. Measurements

### 5.1. Measurement Setup

**Figure 9:**
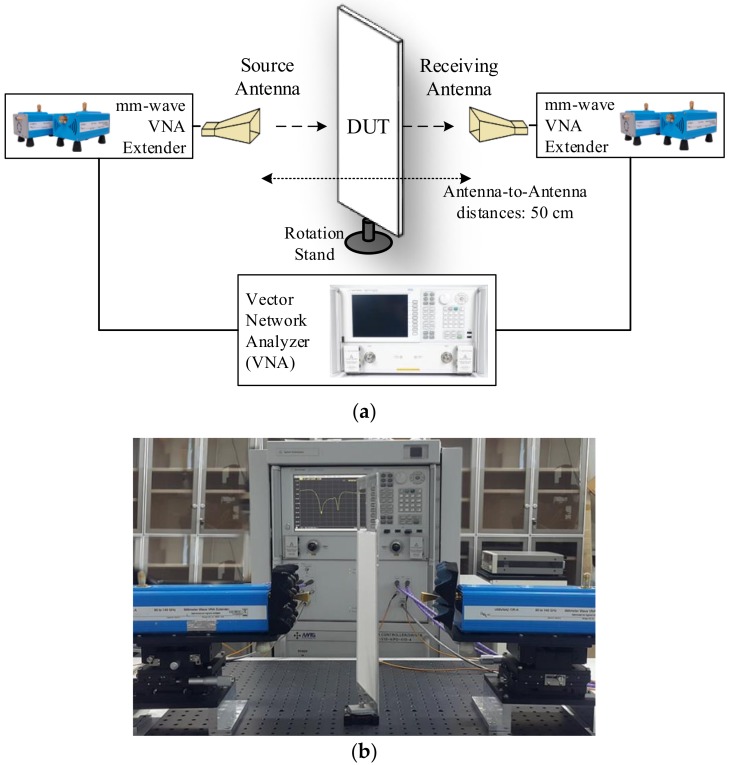
Measurement setup: (**a**) Block diagram, (**b**) DUT with oblique angle incidence of 14°.

As to measure performances of the square- and circular-loop FSS filters, the FSSs were placed in the middle of horn antennas and an incident plane wave were applied using a vector network analyzer. The performances of both FSSs were measured over the frequency range from 90 to 125 GHz, for which two VNA extender mixers and two horn antennas were also used. A device under test (DUT) was mounted on a rotatable part to measure the oblique angle performance. The antenna-to-antenna distance was kept as 50 cm throughout the measurements and the distance from one antenna to the DUT was 25 cm. Figure 9 shows the block diagram and actual measurement setup of the FSS filters.

### 5.2. Measured Responses

**Figure 10:**
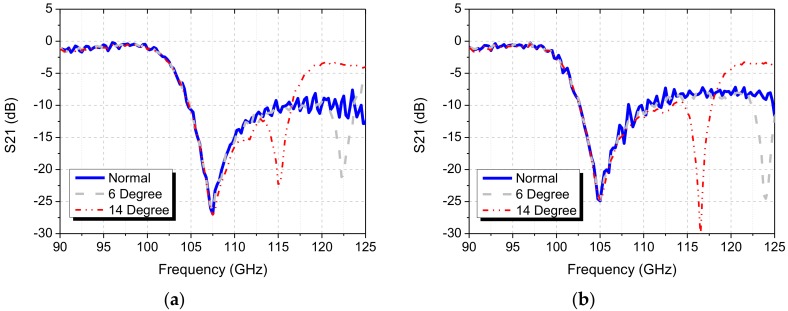
Measured angular performance: (**a**) Square-loop FSS Filter, (**b**) Circular-loop FSS filter.

The measured responses of the single-layer double-sided square-loop and circular-loop FSS filters are shown in Figure 10a,b. For the final design, the dimension parameters were optimized to resonant at 103 GHz for the square-loop FSS and at 104.5 GHz for the circular-loop FSS in the simulation taking into account of the fabrication tolerances. From Figure 10a, it is observed that the square-loop FSS exhibits the rejection of 26.4 dB at the resonant frequency of 107.5 GHz, which is around 4.5 GHz shifted from the simulated performance. On the other hand, in Figure 10b, it is observed that the circular-loop FSS exhibits the rejection of 25.4 dB at the resonant frequency of 105 GHz, which is just 0.5 GHz shifted from the simulated result. Therefore, it is proven with the actual implementation that the circular-loop FSS is more predictable and less sensitive to its dimension parameter errors as compared to the square-loop FSS.

Also, the proposed FSS filters exhibit insensitive response to the oblique incident angle of 14 degrees, which is adequate for the mm-wave imaging diagnostic system application at KSTAR in Daejeon, Korea. The oblique incident angular performance of the proposed FSS can be increased up to 40 degrees by decreasing the period *p* of unit cell or gap *g* between the adjacent elements [6]. In the implemented design, a trade-off between a high Q-factor and high oblique incident performance has been made. In the designed parameters, the large *p* or *g* was selected due to the requirement of the high Q-factor, which consequently limited the oblique incident performance. The high Q-factor can also be achieved by selecting small *w* but the smallest possible *w* was already chosen due to fabrication limitations. For the KSTAR application, the operation frequency range of the proposed FSS was from 90 GHz to 110 GHz (low-pass band region).

### 5.3. Application in Imaging Diagnosis System

**Figure 11:**
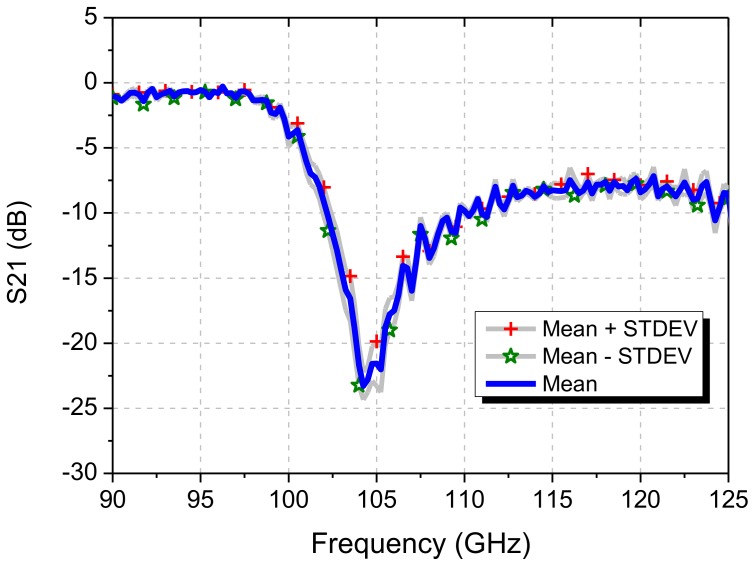
Performance variation of the multiple FSS filter prototypes.

The proposed 105 GHz circular-loop FSS has been implemented and used as a component in the ECEI (Electron Cyclotron Emission Imaging) system, for protecting the imaging antenna-detector array from a high-power stray EM signal. To provide the best performing FSS filters, several filters of the same design were fabricated and tested under same test environment. Figure 11 shows the measured frequency responses of eight circular-loop FSS filters for the KSTAR ECEI system, including the performance deviations with arithmetic mean and standard deviation (STDEV). The measured results of the fabricated filters have negligible differences of ~1 dB in notch-rejection and shift of ~0.25 GHz in resonant frequency due to fabrication tolerances. The proposed circular-loop FSS filter is practical and adequate for the mm-wave imaging diagnostic system applications.

## 6. Conclusions

In this paper, a practical design method of high-performance square-loop and circular-loop FSS filters for use of mm-wave imaging applications is presented. The filters consist of periodic loop structures fabricated on both sides of the thin dielectric substrate. The effects of design parameters on the performance of the FSS filters are discussed and analyzed. Also, a comprehensive comparative study on the performance of square- and circular-loop FSS structures at mm-wave frequency range in the presence of fabrication tolerances is presented. With a high-accuracy fabrication, the square-loop and circular-loop FSS filters perform equivalently. In the presence of appreciable fabrication tolerances, however, it is concluded that the circular-loop based FSS filter produces more consistent, predictable and accurate results. As a design example, square-loop and circular-loop FSS filters resonating at 105 GHz were deigned, fabricated and measured. The circular-loop FSS filter resulted in more accurate and consistent performances and is proved to be a useful configuration when a large-size FSS structure in required, for example, for protection of sensitive receiver electronics of mm-wave imaging systems.

## Figures and Tables

**Table 1:** Comparison of the designed and fabricated parameter values.

	Parameter	Designed Value (mil)	Fabricated Value (mil)	Error (%)
Loop length “*d*”	*d_s_* (square)	24	23.693~23.606	1.46
	*d_c_* (circular)	27	27.098~26.941	0.07
Loop width “*w*”	*w_s_* (square)	04	3.783~3.724	6.16
	*w_c_* (circular)	04	4.266~4.108	4.68
Gap between the loops “*g*”	*g_s_* (square)	36	36.429~36.279	0.98
	*g_c_* (circular)	33	33.244~33.008	0.38

## References

[1] Munk B.A. (2000). Frequency Selective Surfaces: Theory and Design.

[2] Anderson I. (1975). On the theory of self-resonant grids. Bell Syst. Tech. J..

[3] Marcuvitz N. (1951). Waveguide Handbook.

[4] Sung G.H.-H., Sowerby K.W., Neve M.J., Williamson A.G. (2006). A Frequency-selective wall for interference reduction in wireless indoor environments. IEEE Antennas Propag. Mag..

[5] Sarabandi K., Behdad N. (2007). A frequency selective surface with miniaturized elements. IEEE Trans. Antennas Propag..

[6] Mohyuddin W., Woo D.S., Choi H.C., Kim K.W. (2018). A practical double-sided frequency selective surface for millimeter-wave applications. Rev. Sci. Instrum..

[7] Agrawal V., Imbriale W. (1979). Design of a dichroic cassegrain subreflector. IEEE Trans. Antennas Propag..

[8] Wu T.K. (1994). Four-band frequency selective surface with double-square-loop patch elements. IEEE Trans. Antennas Propag..

[9] Costa F., Monorchio A. (2012). A frequency selective radome with wideband absorbing properties. IEEE Trans. Antennas Propag..

[10] Costa F., Amabile C., Monorchio A., Prati E. (2011). Waveguide dielectric permittivity measurement technique based on resonant FSS filters. Microw. Wirel. Compon. Lett..

[11] Gagnon N., Petosa A., McNamara D.A. (2013). Research and development on phase-shifting surfaces (PSSs). IEEE Antennas Propag. Mag..

[12] Yin J.Y., Wan X., Ren J., Cui T.J. (2017). A circular polarizer with beamforming feature based on frequency selective surfaces. Sci. Rep..

[13] Euler M., Fusco V., Cahill R., Dickie R. (2010). 325 GHz single layer sub-millimeter wave FSS based split slot ring linear to circular polarization convertor. IEEE Trans. Antennas Propag..

[14] Li W., Xia S., He B., Chen J., Shi H., Zhang A., Li Z., Xu Z. (2016). A reconfigurable polarization converter using active metasurface and its application in horn antenna. IEEE Trans. Antennas Propag..

[15] Maier T., Bruckl H. (2009). Wavelength-tunable microbolometers with metamaterial absorbers. Opt. Lett..

[16] Kim H.K., Lee D., Lim S. (2016). A fluidically tunable metasurface absorber for flexible large-scale wireless ethanol sensor applications. Sensors.

[17] Lee Y., Kim S.-J., Park H., Lee B. (2017). Metamaterials and metasurfaces for sensor applications. Sensors.

[18] Bayatpur F., Sarabandi K. (2010). Miniaturized FSS and patch antenna array coupling for angle-independent, high-order spatial filtering. Microw. Wirel. Compon. Lett..

[19] Zhang B., Zhang Y., Duan J., Zhang W., Wang W. (2016). An omnidirectional polarization detector based on a metamaterial absorber. Sensors.

[20] Kuznetsov S.A., Paulish A.G., Gelfand A.V., Lazorskiy P.A., Fedorinin V.N. (2011). Bolometric THz-to-IR converter for terahertz imaging. Appl. Phys. Lett..

[21] Chen H.T., Padilla W.J., Cich M.J., Azad A.K., Averitt R.D., Taylor A.J. (2009). A metamaterial solid state terahertz phase modulator. Nat. Photonics.

[22] Meiden H.J. (1999). Application of band-stop filters for the 30-200 GHz range in oversized microwave systems. Rev. Sci. Instrum..

[23] Shen Z., Ito N., Liang Y., Lin L., Domier C.W., Johnsaon M., Luhmann N.C., Mase A., Sakata E. (2017). Protection filters in ecei systems for plasma diagnostics. Plasma Fusion Res..

[24] Hu X., Domier C.W., Luhman N.C. Frequency selective surface applications in millimeter wave imaging diagnostics for fusion plasmas. Proceedings of the 40th International Conference on Infrared, Millimeter, and Terahertz waves (IRMMW-THz).

[25] Kim D.H., Mohyuddin W., Woo D.S., Choi H.C., Kim K.W. (2017). Design of a 75–140 GHz high-pass printed circuit board dichroic filter. Rev. Sci. Instrum..

[26] Yun G.S., Lee W., Choi M.J., Lee J., Kim M., Leem J., Nam Y., Choe G.H., Park H.K., Park H. (2014). Quasi 3D ECE imaging system for study of MHD instabilities in KSTAR. Rev. Sci. Instrum..

[27] Oh J., Cho S., Lee C., Kim J., Choi B. The fabrication of film-type frequency selective surface (FSS) attachable to window glass using inkjet printing technique. Proceedings of the 2008 URSI General Assembly and Scientific Symposium.

[28] Ebrahimi A., Nirantar S., Withayachumnankul W., Bhaskaran M., Sriram S., Al-Sarawi F., Abbott D. (2015). Second-order terahertz bandpass frequency selective surface with miniaturized elements. IEEE Trans. Terahertz Sci. Technol..

[29] Ebrahimi A., Shen Z., Withayachumnankul W., Al-Sarawi S.F., Abbott D. (2016). Varactor-tunable second-order bandpass frequency-selective surface with embedded bias network. IEEE Trans. Antennas Propag..

[30] Huang J., Wu T.-K., Lee S.-W. (1994). Tri-band frequency selective surface with circular ring elements. IEEE Trans. Antennas Propag..

[31] Dewani A.A., Thiel D.V., O’Keefe S.G., Galehdar A. (2015). Optically transparent frequency selective surfaces on flexible thin plastic substrates. AIP Adv..

